# A Metabolomic Investigation of Eugenol on Colorectal Cancer Cell Line HT-29 by Modifying the Expression of APC, p53, and KRAS Genes

**DOI:** 10.1155/2021/1448206

**Published:** 2021-11-18

**Authors:** Elham Ghodousi-Dehnavi, Reza H. Hosseini, Mohammad Arjmand, Sima Nasri, Zahra Zamani

**Affiliations:** ^1^Department of Biology, Faculty of Science, Payame Noor University, Tehran, Iran; ^2^Department of Biochemistry, Pasteur Institute of Iran, Pasteur Avenue, Tehran, Iran

## Abstract

Colorectal cancer is one of the most lethal cancers with a high mortality rate. Chemotherapy results in drug resistance in some cases; hence, herbal medicines are sometimes used in adjunct with it. Eugenol has been reported to have anti-inflammatory, antioxidant, and anticancer properties. Metabolomics is a study of metabolic changes within an organism using high-throughput technology. The purpose of this research was to investigate the anticancer effects of eugenol and variations in p53, KRAS, and APC gene expression and metabolic changes associated with the abovementioned gene expressions using ^1^HNMR spectroscopy. The MTT method was used to determine cell viability and its IC50 detected. After treating HT-29 cells with IC50 concentration of eugenol, RNA was extracted and cDNA was obtained from them and the expression of p53, KRAS, and APC genes was measured using the qRT-PCR technique. Metabolites were extracted using the chloroform-ethanol method, lyophilized, and sent for ^1^HNMR spectroscopy using the 1D-NOESY protocol. Chemometrics analysis such as PLS-DA was performed, and differentiated metabolites were identified using the Human Metabolome Database. Integrated metabolic analysis using the metabolites and gene expression was performed by the MetaboAnalyst website. The observed IC50 for eugenol was 500 *μ*M, and the relative expression of APC and p53 genes in the treated cells increased compared to the control group, and the expression of KRAS oncogene gene decreased significantly. The crucial changes in convergent metabolic phenotype with genes were identified. The results indicate that eugenol exhibits its antitumor properties by targeting a specific biochemical pathway in the cell's metabolome profile due to changes in genes involved in colon cancer.

## 1. Introduction

Colorectal cancer is the third most diagnosed cancer and the second leading cause of cancer mortality worldwide, with a higher prevalence in women [1]. It is a heterogeneous disease due to multiple genetic and molecular changes along with upstream interactions, often resulting in pathological lesions in the large intestinal mucosa. Cancer progression is associated with oncogenic mutations and tumor suppressor genes such as APC, KRAS, and p53, which ultimately lead to metabolic disorders [2].

Despite numerous treatments such as advanced surgery and combination methods such as chemotherapy and radiotherapy, the 5-year survival rate of patients rarely reaches over 60%. Chemotherapy is one of the most important strategies against this cancer, but it has been challenged due to numerous side effects, including drug resistance. Understanding the molecular functions of cancer and the use of natural resources such as plants and their derivatives can be considered a new solution in the prevention and treatment of this cancer [3].

Eugenol, 2-methoxy-4-(2-propenyl) phenol, is a chemical compound found in honey and essential oils of *Syzigium aromaticum* (clove), basil, bay leaf, and other aromatic spices. Eugenol is widely used in traditional Asian medicine due to its medicinal and biological properties including antioxidant, anti-inflammatory, antispasmodic, and antibacterial properties [4]. Eugenol demonstrates both antioxidant and prooxidant properties and modulates the expression of genes implicated in cell growth, apoptosis, and angiogenesis in various cancers, including prostate, colon, liver, and ovarian cancers which prevents cancer progression [5].

Cancer cells need significant metabolic changes to tolerate the growth and progression of cancer, which is mainly related to changes in gene expression patterns in cancer cells, and therefore, the metabolism of cancer cells is significantly different from that of normal cells. Examination of cancer cell metabolite profiles by metabolomics is an effective way to investigate cancer function as it identifies modified pathways that alter metabolites that are involved in the development, progression, and inhibition of cancer [6, 7].

Metabolomics is a powerful downstream technique following proteomics, transcriptomics, and genomics which measures endogenous metabolic material in response to internal and external changes throughout the body. Two throughput technologies, mass spectrometry (MS) and nuclear magnetic resonance spectroscopy (1HNMR), are used in metabolomics, while the latter is a nondestructive and noninvasive method compare to MS. Following NMR spectroscopy, multivariate statistical classification analysis such as partial least discrimination analysis (PLS-DA) is widely used to identify the differentiating metabolites in NMR spectra [8].

There have been earlier reports on the effect of eugenol on colorectal cells, but we decided to carry out an integrative study using ^1^HNMR metabolomics and the expression of the APC and p53 tumor suppressor genes and K-RAS oncogene gene with eugenol on HT-29 adenocarcinoma cells.

## 2. Materials and Methods

Reagents: eugenol and trypan blue solution, 3-(4,5-dimethyl-2-thiazolyl)-2,5-diphenyl-2H-tetrazolium bromide (MTT), and dimethyl sulfoxide (DMSO) were purchased from Sigma-Aldrich (Sigma-Aldrich, USA). RNX plus (Sinagen, Iran), the 2-step real-time-PCR kit (Aldrich-sigma, UK), Dulbecco's Modified Eagle's Medium (DMEM), and the Roswell Park Memorial Institute (RPMI) medium were purchased from Merck KGaA© (Darmstadt, Germany).

### 2.1. Cell Viability Assay Using MTT

#### 2.1.1. Cell Culture

The epithelial HT-29 colorectal cancer cell line (NCBI code C154 and ATCC number HTB-38) was obtained from the Cell Bank of Pasteur Institute of Iran. The cells are routinely karyotyped and examined for mycoplasma contamination. The cells were defrosted under sterile conditions and cultured in DMEM medium containing 10% FBS (bovine fetal serum) and 1% v/v c penicillin-streptomycin and incubated at 37°C with 5% CO_2_ and 95% humidity. The culture medium was changed every 3 days, and when the cell density at the bottom of the flask reached 80%, trypsin/EDTA solution was added to detach them and they were divided into two flasks after centrifugation at 5000*g* at 4°C.

#### 2.1.2. MTT Method

The epithelial colorectal cell line H-29 response to different dilutions of eugenol was measured by the ability of the viable cells' oxidoreductase enzymes to convert the tetrazolium dye 3-(4,5-dimethylthiazol-2-yl)-2,5-diphenyl tetrazolium bromide (MTT) to its insoluble formazan form. The cells were seeded in 96-well plates (1 × 10^4^ cells/well) and incubated for 24 h. After the cells adhered to the bottom of the plate, different dilutions of eugenol (prepared from Sigma-Aldrich) were prepared in the following concentrations using methanol (0, 100, 200, 300, 400, 500, 600, and 700 *μ*M) and cells counted at intervals of 24, 48, and 72 hours incubation at 37°C. The viability of the cells was assessed by trypan blue counting under a phase-contrast microscope using a hemocytometer slide. Then, 4 × 10^4^ cells were added to each well of a 9-well plate to which was added the MTT dye solution (5 mg/ml). After 4 h incubation, the precipitated formazan was dissolved with DMSO and analyzed at 570 nm by using an ELISA reader (BIOTEK U.S.A). Half maximal inhibitory concentration (IC50) was determined in comparison with the control group by plotting the IC50 graph.

#### 2.1.3. Evaluation of APC, KRAS, and p53 Gene Expression after Eugenol Treatment by Quantitative Real-Time Polymerase Chain Reaction (qRT-PCR)

The internal control used was the glycerol aldehyde dehydrogenase housekeeping (GAPDH) gene. The design of the primers and their correct assembly to the relevant and other sequences were carried out by the Blast NCBI site, and primers were purchased from Sinaclon, Iran. The list of primers is given in Table 1. After obtaining the IC50 at 48 hours of eugenol, whole-cell RNA was isolated using the Sinagen plus RNAX kit [9]. The purity and concentration of the extracted RNA were checked using a Nano-Drop ND-1000TM (Thermo Scientific) spectrophotometer at 260/280 nm, and samples displaying 1.8 and 1.9 were examined by electrophoresis on a 1.5% agar gel. The presence of two clear bands 18S and 28S and no fracture or smear on the gel revealed the appropriate quality of RNA which was used in all samples. For cDNA synthesis from mRNA, a 2-step real-time-PCR kit (Aldrich-sigma, UK) with DT oligomer primers and 1 *μ*g of each RNA sample were used for canal synthesis. In order to evaluate the performance of the primers, the melting curve diagram for each gene was examined to confirm the accuracy of the desired gene peak and the lack of dimer primers. The quantitative real-time polymerase chain reaction (qRT-PCR) was performed in 3 replicates [9]. Threshold cycle (CT) for the internal control gene of GAPDH mRNA and CT of the samples was determined, and the relative quantity of mRNA was measured using the relative quality assay of the 2^−ΔΔCT^ method with the following formula:

(1) $$\begin{array}{c} \Delta \Delta \text{CT}=\left({\text{Ct}}^{\text{Target}}-{\text{Ct}}^{\text{GAPDH}}\right)-\left({\text{Ct}}^{\text{Control}}-{\text{Ct}}^{\text{GAPDH}}\right). \end{array}$$

#### 2.1.4. Extraction and Preparation of Samples for ^1^HNMR Spectroscopy

8 × 10^6^ HT-29 cells were collected by centrifugation at 5000 × *g* for 5 min at 4°C after treatment of cells with IC50 dose of eugenol for 48 hours. Nontreated 8 × 10^6^ cells were used as controls in 75 cm^2^ flasks. The temperature throughout the extraction procedure was maintained at 4°C on ice. Cells were washed in 1X PBS and centrifuged as mentioned above and resuspended in 500 *μ*l in a ratio of 2 : 1 of cold methanol and chloroform and put into 1.5 ml Eppendorf tubes; then, 500 *μ*l of water and chloroform were added in a ratio of 1 : 1, and the samples were vortexed. It was sonicated for 10 min on ice and then centrifuged once more at 6000 × *g* for 5 min, and the lower lipophilic phase and upper hydrophilic phase were separated using a Hamilton syringe and transferred to separate vials, and the samples were lyophilized. To prepare the samples for ^1^HNMR, each of the lyophilized hydrophilic samples (*n* = 5) was dissolved in 200 *μ*l of buffer (150 mM potassium phosphate at pH 7.4, 1 mM NaN3, and 0.01% trimethylsilyl propionate (TSP) (Sigma, CA, USA) in 100% D_2_O), and each of the lipophilic lyophilized samples (*n* = 5) was dissolved in 200 *μ*l deuterated chloroform. The samples were then sent to Sharif University to obtain the ^1^HNMR spectrum using the NOESY protocol in a 400 MHz NMR Bruker [10].

#### 2.1.5. Data Analysis

Data obtained from 3 separate experiments for IC50 studies and gene expression were recorded as mean ± standard deviation (SD). Student's *t*-test was used to compare groups in experiments, and *p* value <0.05 was statistically significant. Also, to process the obtained spectra, they were first checked in Mestrec software and the extra water peak 4.7 ppm was removed. The spectra were analyzed by the Prometab program (V.3.3) in MATLAB modeling software. They were converted into Excel matrices and entered into the statistical analysis option of the MetaboAnalyst database to cluster the data PLS-DA, and differentiating chemical shifts are obtained as VIP scores and metabolites identified in the Human Metabolome Database (HMDB). Once more, the detected metabolites were inserted in the joint pathway analysis option for integrative analysis with the PubMed identification number of genes and the most important metabolic pathways affected by the genes and identified.

## 3. Results

### 3.1. The Effect of Eugenol on the Viability of HT29 Colon Cells

The toxicity of eugenol on HT-29 cells was investigated, and the percentage of live cells in eugenol-treated concentrations for 24, 48, and 72 hours is shown in Table 2. Decreased cell viability was seen to be directly related to concentration- and time-dependent IC50 values (concentration leading to 50% survival) which are presented in Table 1 in all the 3 time periods.

### 3.2. Evaluation of APC, KRAS, and p53 Gene Expression after Eugenol Treatment by qRT-PCR

**Figure 1:**
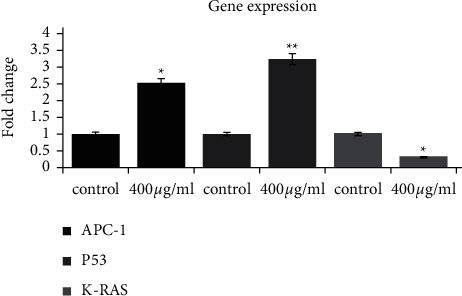
Expression of APC, KRAS, and p53 genes in the HT-29 colorectal cancer cell line treated with (500 *μ*M/ml) eugenol for 48 hours using qRT-PCR. Data are expressed as mean ± standard deviation (^*∗*^*p* < 0.05,  ^*∗∗*^*p* < 0.01).

As shown in Figure 1, the analysis of qRT-PCR data in relation to gene expression by the comparative ΔCT method showed that treatment with IC50 eugenol increased the expression of p53 tumor suppressor gene by 3.2-fold and increased APC expression by 2.5-fold and decreased KRAS oncogene gene by 0.3-fold compared to the control group.

### 3.3. Metabolomics of Raw Spectra of Eugenol on HT-29 Colon Cells

**Figure 2:**
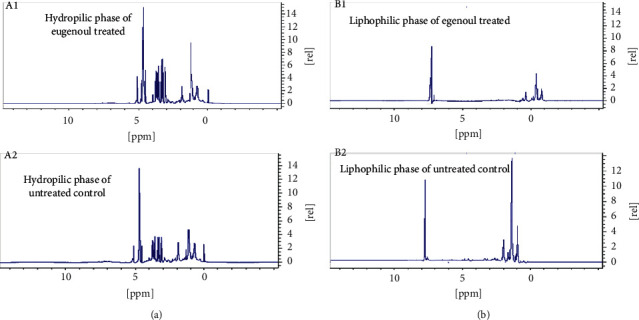
^1^HNMR spectra of (a) the hydrophilic phase of eugenol-treated HT-29 cells (A1) and untreated controls (A2) and (b) the lipophilic phase of eugenol-treated HT-29 cells (B1) and untreated controls (B2).

**Figure 3:**
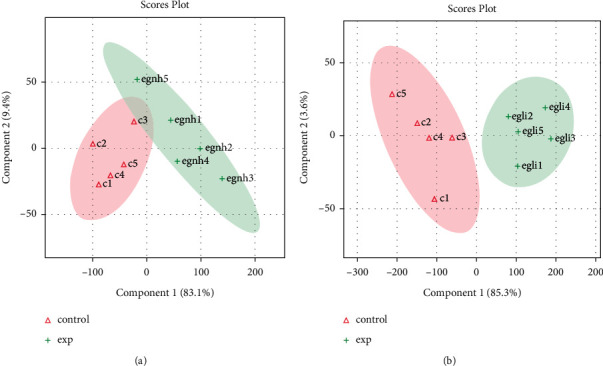
Score plot PLSDA of HT-29 cells treated with eugenol (pink color) and the control group (green color) in the (a) hydrophilic and (b) lipophilic phase.

**Figure 4:**
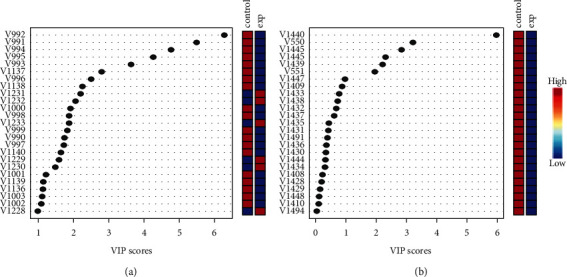
VIP scores of HT-29 cells treated with eugenol and the control group in the (a) hydrophilic phase and (b) lipophilic phase.

**Figure 5:**
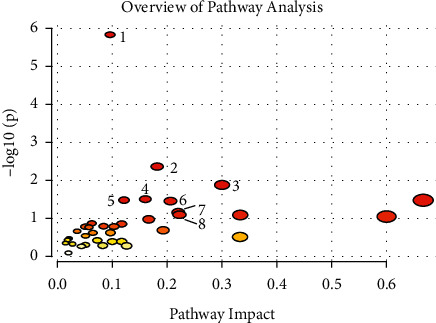
Summary of the joint pathway analysis of metabolites and genes using overrepresentation analysis and a hypogeometric test. Circles higher, bigger, and closer to the *x*-axis are more significant. (1) Aminoacyl-tRNA biosynthesis; (2) valine, leucine, and isoleucine biosynthesis; (3) biotin metabolism; (4) steroid biosynthesis; (5) pantothenate and CoA biosynthesis; (6) glycerolipid metabolism; (7) galactose metabolism; And (8) glutamine and D-glutamate metabolism.

Figures 2(a) and 2(b) depict the raw spectra of the control cells as compared to those treated with IC50 dose of eugenol in both the hydrophilic and lipophilic groups, and the differences are clearly seen. The score plots of PLS-DA are depicted in Figure 3(a) (hydrophilic phase) and Figure 3(b) (lipophilic phase), and a good separation is displayed. The important variables are identified as VIP scores in Figure 4(a) (hydrophilic phase) and Figure 4(b) (lipophilic phase). The integrated analysis demonstrates the important pathways changed by eugenol and the affected genes Figure 5. The impact of the pathways and their *p* values, the metabolites participating in each pathway, and the respective genes involved are presented in Table 3.

## 4. Discussion

One of the most important components of cancer cells is the disruption of growth and proliferation of uncontrolled normal cells that are normally needed to survive [11]. In this study, the cytotoxicity of eugenol on HT-29 colorectal adenocarcinoma cells was first investigated by MTT assay, and it was found that eugenol decreased the viability of these cells in increasing dose- and time-dependent manner. The IC50 value of eugenol was determined at about 500 *μ*M. Earlier studies on HT29 have shown similar IC50 concentrations [12] from 130 to 750 *μ*M on other CRC cell lines [5, 12]. In this regard, other studies on the antiproliferative properties of eugenol in inhibiting cancer cell lines such as placenta, prostate, leukemia, lung, liver, bone marrow, breast, melanoma, submandibular glands, ovarian, and cervical metastases were performed, and the IC50 value for these cells ranged from 0.5 *μ*M in primary melanoma cancer cells to 1600 *μ*M in ovarian metastatic cells corroborating our findings [5, 13–15].

Colorectal cancer is a multistage disease that is characterized by successive changes in many genes, including APC, KRAS, and p53. These lead to variations in several important signaling pathways of colorectal cancer cells such the APC Wnt/*β*-Catenin pathway, KRAS and p53 signaling pathways and causes several biochemical changes, including changes in metabolism for cancer progression and greater energy expenditure [2]. In addition to the fact that genes cause changes in metabolites, it could be that the metabolites themselves may alter the expression of genes involved in cancer. Identification and measurement of metabolite levels lead to disease diagnosis, management, and drug development [6, 7].

In this investigation, the treatment of cells with IC50 concentration of 500 *μ*M caused a significant increase of 3.2-fold in tumor suppressor genes p53 and 2.5-fold augment in APC and 0.3-fold decrease in expression of KRAS oncogene genes. In other studies, eugenol induced apoptosis in skin tumors via the c-Myc pathway, H-ras from the ras oncogene gene family, and by regulating p53 gene expression [13], as well as inhibiting the growth and induction of apoptosis through the p53 gene and associated pathways in colorectal [12] and cervical cancer cells [15]. In eugenol-treated animal models, it inhibited the growth of gastric cancerous tumors by increasing p53 gene expression via the NF-*κ*B pathway [16].

The most important altered metabolic cycles and metabolites integrated with the expression of APC, KRAS, and p53 genes with eugenol treatment are listed in Table 3.

The relevant cycles include the three pathways of t-RNA biosynthesis, the biosynthesis of branched-chain amino acids valine, leucine, and isoleucine, and the metabolism of glutamate and glutamine. Altered metabolites in these pathways include L-glutamate, L-valine, L-isoleucine, L-lysine, L-proline, L-tyrosine, and L-tryptophan. Aminoacyl-tRNA (ARS) enzymes possess an evolved mechanism, and their structures are responsible for charging amino acid-dependent tRNA molecules and are the first step in protein synthesis in the cell. Its structure has a catalytic and a noncatalytic domain called AIMP or scaffold proteins that interacts with various regulatory factors in the cell. In addition to participating in cellular homeostasis, they play an important role in antitumor mechanisms [17]. Any mutation or change in the genes encoding ARS enzymes causes changes in cell function and various diseases. These enzymes are also involved in various processes such as cytokine activity in inflammatory signaling pathways and angiogenesis, cell growth, and proliferation [17, 18]. Tryptophanyl-tRNA synthetase (TrpRS) belongs to the aminoacyl-tRNA synthetase family, in addition to protein synthesis is involved in cytokine activity in inflammatory signaling and angiogenesis pathways, and is induced by IFN*γ* and regulated by the p53 gene, causing the progression and metastasis of colorectal cancer [19].

Other amino acids, such as the branched-chain amino acids BCAA, valine, leucine, and isoleucine also act as major amine donors for the biosynthesis of alanine and glutamine and can supply Krebs cycle organic molecules in a variety of ways. Mutations in the KRAS gene in pancreatic cancer (PDAC) have caused elevated levels of this branched-chain amino acid [20]. In another study, a mutation in ras gene expression caused melanoma cancer cells to become dependent on this branched-chain amino acid [21].

Another important cycle is biotin metabolism in which the modified metabolites in this cycle are biotin and L-lysine. Biotin as a cofactor of four important enzymes of carboxylases is involved in metabolic processes such as the metabolism of fatty acids, glucose, and amino acids and as a factor in the proliferation and growth and improvement of immune system function at the cellular level. The amount of biotin as well as the amount of biotin receptors in tumors and cancer cells such as the colon, lung, and breast is very high [22]. Biotin is mediated by various pathways such as biotinyl-AMP, cGMP, NF-*κ*B, Sp1 with Sp3, and tyrosine kinase receptors that affect gene expression, and most of these pathways are usually associated with the expression of the K-RAS oncogene [23] or the p53 tumor suppressor gene [24].

Steroid biosynthesis is another important pathway which includes cholesterol, cholesterol ester, and lathosterol. Cholesterol, as one of the components of the membrane and also having derivatives with different biological functions, plays a role in the progression of cancer both in terms of proliferation and energy supply. On the other hand, it plays a role in suppressing the immune system in breast, melanoma, ovarian, colorectal, and prostate cancers; its absorption and metabolism are high [25]. In addition, in low-survival cancers such as glioblastoma and melanoma, SREBP target gene expression is activated or reregulated in the path of cholesterol metabolism [26]. Cholesterol and its derivatives, on the other hand, can facilitate migration and tumor signaling pathways by altering membrane junctions. In breast cancer, the concentration of 6-oxo-cholestan-3*β* and 5*α*-diol is high, which binds to the glucocorticoid receptor and intensifies the carcinogenicity in this cancer [27]. Alternatively, it is observed that cancer cells increase the activity of the mevalonate pathway by reprogramming metabolism to counteract cholesterol lowering. Oncogene genes, such as KRAS, increase the activity of this pathway, and conversely, the tumor suppressor gene p53 inhibits this pathway [25]. For example, the p53 gene regulates the transport of ABCA1 cholesterol and by limiting the maturity of SREBP2 and ultimately suppresses the mevalonate pathway [28].

Other important pathways are the biosynthesis of coenzyme A which is derived from pantothenic acid (vitamin B5) and the metabolically active form of pantothenate, which plays a key role in energetic processes such as the oxidation of pyruvate and fatty acids, the metabolism of proteins, and the synthesis of hormones [29]. Acetylation of lysine residues is also carried out with the help of this coenzyme. Increasing the expression of p53 through the NF-*κ*B pathway reduces the number of glucose transducers and inhibits glycolysis and also affects the expression of pyruvate dehydrogenase kinase-2 by negatively regulating pyruvate dehydrogenase and increasing pyruvate to acetyl coenzyme A conversion [6, 30]. The MCT-1 monocarboxylate transporter is a pyruvate transporter and as part of the WNT pathway can modulate pyruvate dehydrogenase kinase expression and relative levels of glycolysis and oxidative phosphorylation [31].

Another important pathway is glycerolipid metabolism in which the two altered metabolites in this pathway include phosphatidate and triacylglycerol. One of the most important metabolic roles of lipids is to partake in the autophagy process by participating in the mTOR complex which is an important target in the cancer cell survival signal. The formation process of this complex begins with the phosphatidylinositol 3-kinases. Important lipids involved in this process are 3-phosphatidyl inositol phosphate, diacylglycerol, and phosphatidate, which are independently involved in the downstream pathway of mTOR signaling [32]. The p53 gene, in addition to normal functions, regulates the two pathways of IGF/AKT-1 and mTOR in the endosomal chamber, which controls metabolism and cell proliferation [33].

Another significant pathway is galactose metabolism. The altered metabolites in this pathway are D-glucose and alpha -D-glucose. Galactose is an important structural element in macromolecules and also is an energy source in cell growth. Disorders in the metabolism of galactose and the accumulation of galactose and its metabolites lead to diseases such as ovarian cancer and its resistance to chemotherapy, especially cisplatin [34]. In a study of HepG2-type liver cancer cells, replacing glucose with glucose in the cell culture medium reduced glycolytic charge and biosynthesis and changed ATP production from substrate surface phosphorylation to mitochondrial OXPHOS [35]. Galactose metabolism was one of the pathways identified in intestinal and fecal mucosa cells in patients with colorectal cancer [36]. Glucose is the main source of cellular energy, especially in cancer cells such as the colorectal, in which the Warburg metabolic phenotype is very evident. Mutation in the p53 gene through different signaling pathways causes more expression of cytoplasmic transporters of GLUT1, 2, 3, and 4 and the inhibition of pyruvate dehydrogenase kinase 2 expression in order to increase glucose consumption and the production of metabolites for proliferation from pathways such as the pentose phosphate pathway to the progression of cancer [2, 6]. Mutations in the KRAS genes also cause changes in enzymes involved in pathways of glucose metabolism such as glucose uptake, amino acid metabolism, and pentose phosphate, as well as the mitochondrial pathway of OXPHOS of colorectal cancer [37]. Inactivation of the APC gene also activates the WNT-B catenin pathway, which increases glycolysis and inhibits the conversion of pyruvate to acetyl coenzyme A [38].

In previous studies, eugenol altered metabolic reprogramming in cancer cells in a variety of ways. Eugenol showed its anticancer properties in oral squamous cell carcinomas (HSC-2) with metabolic changes in the glycolysis pathways, glutamate and amino acid metabolism, and the OXPHOS mitochondrial pathway and regulation of ATP production [39]. Eugenol also showed its anticancer effect in MCF10A-ras breast cancer cells by affecting energy metabolism pathways including reducing fatty acid oxidation and glycolysis cycle and increasing oxidative stress by lowering the regulation of the c-Myc/PGC-1*β*/ERR*α* signaling pathway [40].

## 5. Conclusions

Although data analysis predicted more metabolites and pathways, we selected the pathways according to *p* values and their relationship to the mentioned genes. In this study, the treatment of HT-29 cells with eugenol altered the expression of genes involved in the tumorigenesis process of colorectal cancer and, consequently, the metabolic profile of colorectal cancer cell lines HT-29. It was found that eugenol caused significant metabolic pathways and cancer inhibition by inhibiting tumor suppressor genes APC and p53 and the oncogene gene KRAS. However, for further confirmation, further investigation is required to expand these findings' validity and explore the signaling pathways associated with these changes.

## Figures and Tables

**Table 1:** List of primers, nucleotide sequence, and melting points of primers.

Primer name	Forward 5′ to 3′	Reverse 5′ to 3′	Tm°C	
			Forward	Reverse
P53	GCCCAACAACACCAGCTCCT	CCTGGGCATCCTTGAGTTCC	60.88	60.76
KRAS	CTATTCGCAGCTCACACAGTTTAC	TTCTTAATTTGGTCTGCGGC	60.11	60.18
APC	GACTGGTATTACGCTCAACTTCA	CAATTGCCTTCTGGTCATATCTG	60.05	60.08
GAPDH	AGGGCTGCTTTTAACTCTGG	CCCCACTTGATTTTGGAGGG	59.04	60.27

**Table 2:** Inhibitory effects of the HT-29 cell line treated with eugenol for 24, 48, and 72 hours. This experiment was performed in three replications. Data are presented as mean ± SD of three independent experiments.

Cell survival percentage% ± SD			
Eugenol concentration (*μ*M)	24 hours	48 hours	72 hours
**0**	97/29 ± 1/68^*∗*^	97/04 ± 1/43	96/29 ± 1/01^*∗∗*^
**100**	97/34 ± 2/86	93/83 ± 2/66	86/51 ± 2/58
**200**	95/28 ± 3/18^*∗*^	80/61 ± 1/533	73/81 ± 1/19
**300**	88/06 ± 2/73	73/59 ± 1/48^*∗*^	65/91 ± 2/63
**400**	78/02 ± 1/71	61/18 ± 3/35	53/14 ± 3/15
**500**	66/81 ± 3/28^*∗*^	47/39 ± 3/68	42/80 ± 2/41
**600**	52/04 ± 2/37	36/28 ± 1/16	29/51 ± 1/32^*∗*^
**700**	41/46 ± 1/09	23/43 ± 2/71	17/43 ± 3/23
**IC50**	584/72 ± 5/27	523/64 ± 3/23	451/69 ± 5/09

(^*∗*^*p* < 0.05,  ^*∗∗*^*p* < 0.01).

**Table 3:** Results of the integrated analysis of metabolic cycles and genes from the MetaboAnalyst database and their relationship with potential genetic changes in colorectal cancer.

Metabolic pathway	Participating metabolites	Levels of metabolites	Total no. of compounds in the pathway	Hits actual number of matched numbers	Pathway impact value	Raw *p*	Change in gene expression		
							APC 2-fold>+	P53 3-fold>+	KRAS 0.3<−
Aminoacyl-tRNA biosynthesis	L-glutamate L-valine L-isoleucine L-lysine L-proline L-tyrosine L-tryptophan	↓	74	7	0.09589	1.4684*E* − 6	✓	✓	✓
		↓							
		↓							
		↓							
		↓							
		↓							
		↓							
		↓							
Valine, leucine, and isoleucine biosynthesis	L-valine L-isoleucine	↓	12	2	0.18182	0.0044219	—	—	✓
		↓							
Biotin metabolism	L-lysine Biotin	↓	21	2	0.3	0.013428	—	✓	✓
		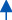							
Steroid biosynthesis	Lathosterol Cholesterol Cholesterol ester	↓	82	3	0.16049	0.031442	—	✓	✓
		↓							
		↓							
Pantothenate and CoA biosynthesis	L-valine Pantothenate	↓	34	2	0.12121	0.033545	✓	✓	—
		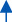							
Glycerolipid metabolism	Phosphatidate Triacylglycerol	↓	35	2	0.20588	0.035395	—	✓	—
		↓							
Galactose metabolism	alpha-D-Glucose D-glucose	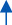	51	2	0.22	0.06987	✓	✓	✓
		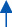							
D-glutamine and D-glutamate metabolism	L-glutamate	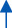	10	1	0.33333	0.082955	—	✓	✓

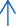
 shows increase and 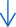 shows decrease of the metabolite level. ✓ shows involvement of genes in the cycle.

## Data Availability

Data can be made available on request.
